# Effect of total cholesterol and statin therapy on mortality in ARDS patients: a secondary analysis of the SAILS and HARP-2 trials

**DOI:** 10.1186/s13054-023-04387-9

**Published:** 2023-03-28

**Authors:** Shaun M. Pienkos, Andrew R. Moore, Jiazhen Guan, Joseph E. Levitt, Michael A. Matthay, Rebecca M. Baron, John Conlon, Daniel F. McAuley, Cecilia M. O’Kane, Angela J. Rogers

**Affiliations:** 1 Department of Medicine, Division of Pulmonary and Critical Care Medicine, Stanford University, 300 Pasteur Dr H3143, Stanford, CA 94305 USA; 2 Division of Pulmonary & Critical Care, Brigham and Women’s Hosp, 15 Francis Street, Boston, MA 02115 USA; 3 Department of Medicine, Cardiovascular Research Institute, University of California, 35 Medical Center Way, San Francisco, CA 94143 USA; 4 Wellcome-Wolfson Institute for Experimental Medicine, Queen’s University, 97 Lisburn Road, Belfast, BT9 7BL UK; 5 Regional Intensive Care Unit, Royal Victoria Hospital, 274 Grosvenor Rd, Belfast, BT12 6BA UK

**Keywords:** Acute respiratory distress syndrome, Sepsis, Statins, Cholesterol, Critical care

## Abstract

**Background:**

Two acute respiratory distress syndrome (ARDS) trials showed no benefit for statin therapy, though secondary analyses suggest inflammatory subphenotypes may have a differential response to simvastatin. Statin medications decrease cholesterol levels, and low cholesterol has been associated with increased mortality in critical illness. We hypothesized that patients with ARDS and sepsis with low cholesterol could be harmed by statins.

**Methods:**

Secondary analysis of patients with ARDS and sepsis from two multicenter trials. We measured total cholesterol from frozen plasma samples obtained at enrollment in Statins for Acutely Injured Lungs from Sepsis (SAILS) and Simvastatin in the Acute Respiratory Distress Syndrome (HARP-2) trials, which randomized subjects with ARDS to rosuvastatin versus placebo and simvastatin versus placebo, respectively, for up to 28 days. We compared the lowest cholesterol quartile (< 69 mg/dL in SAILS, < 44 mg/dL in HARP-2) versus all other quartiles for association with 60-day mortality and medication effect. Fisher’s exact test, logistic regression, and Cox Proportional Hazards were used to assess mortality.

**Results:**

There were 678 subjects with cholesterol measured in SAILS and 509 subjects in HARP-2, of whom 384 had sepsis. Median cholesterol at enrollment was 97 mg/dL in both SAILS and HARP-2. Low cholesterol was associated with higher APACHE III and shock prevalence in SAILS, and higher Sequential Organ Failure Assessment score and vasopressor use in HARP-2. Importantly, the effect of statins differed in these trials. In SAILS, patients with low cholesterol who received rosuvastatin were more likely to die (odds ratio (OR) 2.23, 95% confidence interval (95% CI) 1.06–4.77, *p* = 0.02; interaction *p* = 0.02). In contrast, in HARP-2, low cholesterol patients had lower mortality if randomized to simvastatin, though this did not reach statistical significance in the smaller cohort (OR 0.44, 95% CI 0.17–1.07, *p* = 0.06; interaction *p* = 0.22).

**Conclusions:**

Cholesterol levels are low in two cohorts with sepsis-related ARDS, and those in the lowest cholesterol quartile are sicker. Despite the very low levels of cholesterol, simvastatin therapy seems safe and may reduce mortality in this group, though rosuvastatin was associated with harm.

**Graphical abstract:**

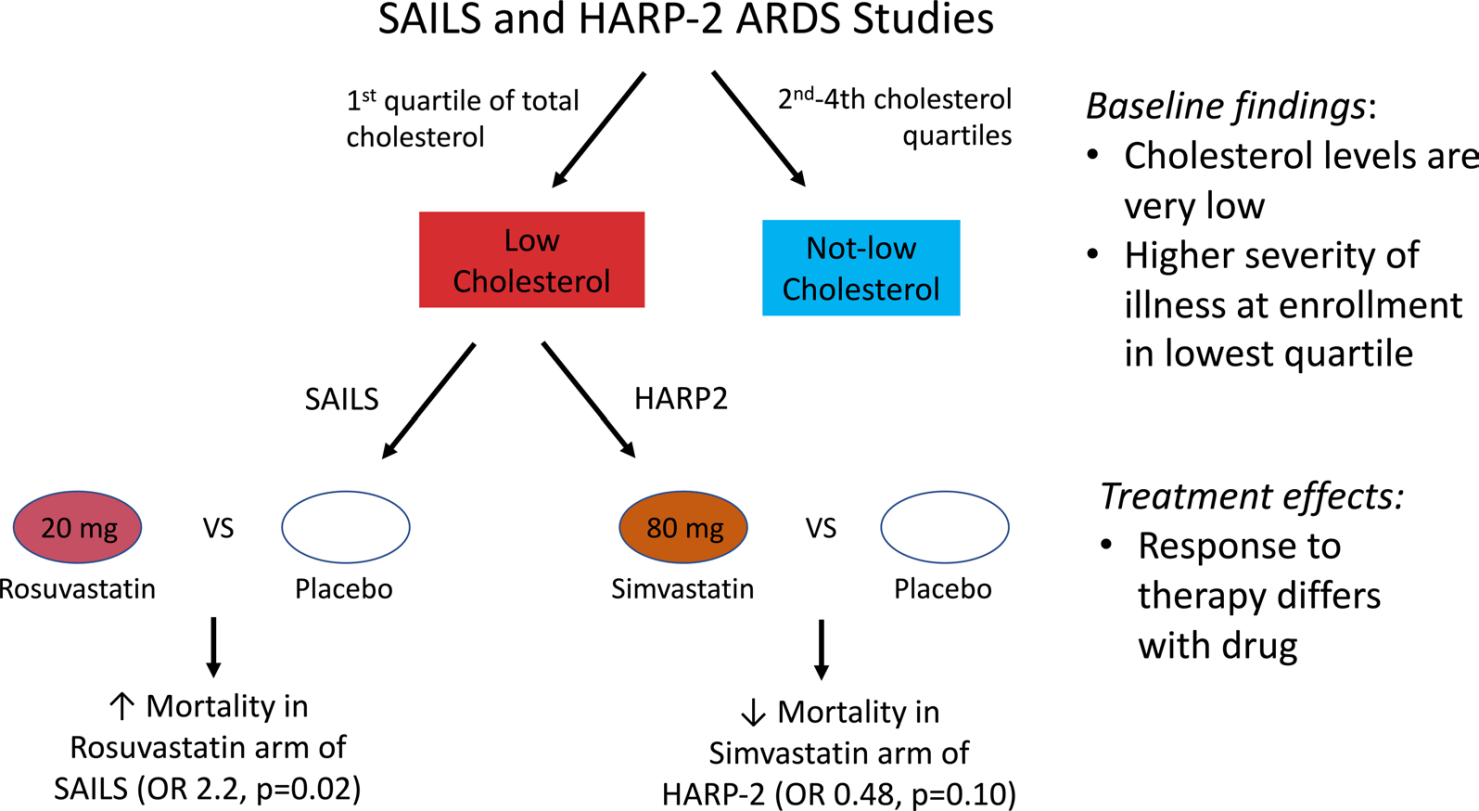

**Supplementary Information:**

The online version contains supplementary material available at 10.1186/s13054-023-04387-9.

## Background

Acute respiratory distress syndrome (ARDS) is a heterogeneous condition with high mortality and lacks targeted medical therapies [1, 2]. Hydroxymethyl-glutaryl coenzyme A reductase inhibitors (statin medications) have been investigated in ARDS and other critical illness due to associations with decreased inflammatory markers, improved outcomes in infection [3], and less organ dysfunction [4]. Two large clinical trials have assessed the efficacy of simvastatin (HARP-2 trial) and rosuvastatin (SAILS trial) in improving ARDS outcomes. Both trials showed no overall benefit, and statins were associated with greater incidence of non-serious adverse events [5, 6]. However, a secondary analysis of the HARP-2 trial showed that subjects with a hyper-inflammatory subphenotype demonstrated survival benefit in those who received simvastatin [7]. In contrast, a benefit was not seen with rosuvastatin in the hyper-inflammatory subphenotype in the SAILS trial [8]. Subgrouping of subjects in the SAILS trial by baseline plasma IL-18 levels and an increase in IL-18 levels over 3 days in the study suggested that patients with inflammasome activation (as measured by high IL-18 levels) in the rosuvastatin administration group had higher mortality [9]. These studies support the value of secondary analyses to propose subgroups which are most likely to benefit from statins in ARDS.

Interestingly, the effect of statin medications on lipid profiles as it pertains to outcomes in critical illness has not been well studied. Cholesterol has proposed roles in normal lung physiology, including its integration into surfactant and delivery of the antioxidant Vitamin E to type II pneumocytes [10, 11]. In disease states, cholesterol additionally appears to have important functions. Oxidized LDL has been shown to bind toll-like receptor 4, thus upregulating innate immunity [12]. Cholesterol-containing lipoproteins bind and neutralize lipopolysaccharide (LPS), the cell membrane component of gram-negative bacteria which causes inflammation through the innate immune system [13], thereby offering a potentially protective mechanism in sepsis. Thus, some have hypothesized that low peripheral blood cholesterol levels might correlate with worse outcomes in critical illness.

Cholesterol synthesis is downregulated in critical illness by multiple mechanisms, including pro-inflammatory cytokines which affect transcription of cholesterol products [14], and alterations in reverse cholesterol transport may also be important [15]. Low total cholesterol levels have been reported in multiple cohorts with systemic inflammatory response syndrome and sepsis. [16] Hypocholesterolemia in surgical and critically ill patients is a poor prognostic factor which often mirrors illness severity and trajectory [17]. Due to the physiologic roles of cholesterol and the lipid lowering effects of statins, it is possible that administering these medications may be inappropriate for use in critically ill patients with cholesterol levels which are already depressed.

The present study investigates the effect of total cholesterol levels on response to statin therapy in ARDS using data from the SAILS and HARP-2 trials. First, we hypothesized that subjects with lower baseline cholesterol would have higher mortality than those with higher cholesterol, and second that randomization to statin therapy could be harmful in this high-risk subgroup.

## Methods

### Population

This is a secondary analysis of randomized clinical trial data from the ARDS Network Statins for Acutely Injured Lungs from Sepsis (SAILS) trial [6] and the Hydroxymethylglutaryl-CoA reductase inhibition with simvastatin in Acute lung injury to Reduce Pulmonary dysfunction (HARP-2) trial [5]. The SAILS trial enrolled 745 patients with ARDS from sepsis from 2010 to 2013 at 44 hospitals. Subjects were randomized 1:1 to receive rosuvastatin (40 mg loading dose followed by 20 mg daily) or placebo for up to 28 days. The HARP-2 trial enrolled 540 subjects at 40 hospitals who developed ARDS from any cause from 2010 to 2014. Among the 540 subjects, 384 had “pneumonia” or “sepsis” as a cause of ARDS and are included in the primary analysis of patients with sepsis. Subjects were randomized in a 1:1 ratio to receive simvastatin 80 mg or placebo once daily for up to 28 days. In both trials, subjects were eligible for enrollment for 48 h after ARDS onset. The SAILS (ClinicalTrials.gov Identifier: NCT00979121, accepted September 16, 2009) and HARP-2 (International Standard Registered Clinical Trial Number: 88244364, approved August 9, 2010) trials were prospectively registered. Details of the study designs can be found in the original publications [5, 6].

### Cholesterol measurement

Total cholesterol levels were obtained from frozen plasma samples which were collected at the time of enrollment in the clinical trials. Total cholesterol was measured in the year 2016 for SAILS samples using the Infinity Cholesterol Liquid Stable Reagent (TR2242, from ThermoFisher), and the Amplex Red Cholesterol Assay (A12216, from ThermoFisher) was used for HARP-2 samples in 2020.

### Statistical analysis

To test our hypothesis that mortality would be higher in subjects with lower cholesterol who received a statin, individuals were assigned to a “low cholesterol” or “not-low cholesterol” group defined by lowest quartile of total cholesterol versus the remaining three quartiles. To our knowledge, there is no universally accepted definition of low total cholesterol. In 2018, the global age-standardized mean total cholesterol was 178 mg/dL (95% confidence interval 174–182 mg/dL) for women and 172 mg/dL (95% confidence interval 168–176 mg/dL) for men [18]; however, these sample means are unlikely to apply to critically ill patients. Because our hypothesis was that very low cholesterol may be deleterious to multiple biological processes, we used quartiles to inform relative differences within these cohorts. Data from the SAILS and HARP-2 studies were analyzed separately due to the possibility that there are differences in ARDS mortality impact between rosuvastatin and simvastatin, [7, 8] there are phenotypic differences in the two trials at baseline, and because different cholesterol assays were used for each trial. The lowest cholesterol quartile is defined by < 69 mg/dL in SAILS, and < 44 mg/dL in HARP-2.

Our primary outcome was mortality at 60 days in subjects with sepsis-induced ARDS (the primary outcome in SAILS). We first compared the lowest cholesterol quartile versus all other quartiles in both trials. Available illness severity indices at enrollment were compared using the Wilcoxon rank-sum test. The illness severity score for SAILS was APACHE III, while HARP-2 reported APACHE II and SOFA scores. Mortality at 60 days for those randomized to a statin was compared using Fisher’s exact test. We also compared mortality for subjects in the lowest quartile randomized to statin versus placebo using Fisher’s exact test. Sixty-day mortality was evaluated using logistic regression which included cholesterol (lowest quartile vs. others), randomization to statin, and their interaction term, controlling for age, gender and body mass index (BMI). These covariates were chosen because of impact on total cholesterol levels in general population data [18, 19]. Illness severity scores were not included in our initial regression models; however, we performed regression with APACHE II/III score as a post-hoc analysis shown in Table 2. We used Kaplan–Meier survival analysis and the Cox Proportional Hazards model (variables included age, gender, BMI, low cholesterol status, randomization to statin or placebo, and the interaction between cholesterol and treatment arm) to evaluate survival for 60 days following randomization.

The secondary outcomes included the same Fisher’s exact tests, survival analysis and logistic regression for mortality at 60 days described above, but applied to the entire HARP-2 cohort (given that HARP-2 enrolled subjects with non-sepsis etiologies of ARDS in contrast to only subjects with sepsis-induced ARDS in both trials for the primary outcome). Additionally, we examined the clinical characteristics of low cholesterol in subjects randomized to receive placebo in both trials. Wilcoxon rank-sum tests and Fisher’s exact tests were used for continuous and categorical variables, respectively. The placebo group was used to remove any effect of statin therapy on survival, instead investigating the impact of cholesterol level alone. We assessed mortality at 60 days for patients in the lowest cholesterol quartile versus the top three quartiles using Fisher’s exact test. To increase statistical power, we also evaluated 60-day mortality for all subjects in both trials with cholesterol levels available.

A sensitivity analysis was conducted with several additional thresholds for low cholesterol, and these results are presented in Additional file 1: Table S2. Association of illness severity scores (APACHE II, APACHE III, and SOFA), shock/vasopressor use, and 60-day mortality were evaluated against these alternative cholesterol cutoffs. The R package “cutpointr” was used to determine optimal cholesterol threshold for predicting 60-day mortality with maximal sensitivity and specificity [20].

All data analysis was completed with R Studio, Version 1.2.

## Results

### Population

Of the 745 subjects enrolled in SAILS, 678 subjects had sufficient plasma samples available to obtain baseline total cholesterol levels, and thus were included in this analysis. In the SAILS trial, the predominant cause of sepsis-associated ARDS was pneumonia, with the remaining cases largely consisting of other infections and aspiration. Median total cholesterol was low in this cohort, 97 mg/dL (IQR 69–130), with the lowest quartile defined by total cholesterol < 69 mg/dL.

Our analysis of the HARP-2 cohort included 509 subjects from whom cholesterol levels were attainable, including 384 subjects with sepsis. The most common etiologies of ARDS in the entire cohort were identified as pneumonia and sepsis. Median total cholesterol levels for all patients in HARP-2 were also low, 97 mg/dL (IQR 44–168), with the lowest quartile defined by total cholesterol < 44 mg/dL. These values were not significantly different when calculated for all 509 HARP2 subjects versus only the 384 with sepsis (data not shown).

### Association of low cholesterol with illness severity and mortality

As shown in Table 1, low baseline cholesterol was associated with several indicators of higher illness severity. In the SAILS cohort, APACHE III scores were higher (mean 102 (95% CI 98.0–106.8) vs. 90.8 (95% CI 88.3–93.3), *p* < 0.001) and shock was more prevalent (OR 2.13, 95% CI 1.43–3.20, *p* < 0.001). Mortality did not significantly differ by baseline cholesterol (28.8% vs 26.6%, *p* = 0.61). In HARP-2, APACHE II scores did not differ, but low cholesterol group had higher SOFA (mean 9.94 (95% CI 9.29–10.58) vs. 8.5 (95% CI 8.12–8.88, *p* < 0.001) and vasopressor use at enrollment (85.3% vs. 63.3%, OR 3.34, 95% CI 1.77–6.71, *p* < 0.001). There was a substantial 60-day mortality difference in HARP-2, (42.1% in the low cholesterol group versus 26.6% in the not-low cholesterol group, OR 2.00, 95% CI 1.19–3.33, *p* < 0.01).

**Table 1:** Baseline characteristics of SAILS and HARP-2 cohorts stratified by cholesterol level

Clinical Characteristic	SAILS Low Cholesterol (*n* = 163)	SAILS Not-low Cholesterol (*n* = 515)	HARP-2 Low Cholesterol (*n* = 95)	HARP-2 Not-low Cholesterol (*n* = 289)
Age (years)	55 [44–67]	55 [42–65]	57 [44–66]	53 [42–66]
Female	73 (44.8%)	273 (53.0%)	40 (42.1%)	138 (47.8%)
Illness Severity Scores				
APACHE II			19 [14–23]	18 [15–24]
APACHE III*	103 [82–122]	89 [71–107]		
SOFA**			10 [8–12]	8 [6–10]
Shock/Vasopressors***	94 (57.7%)	213 (41.4%)	81 (85.3%)	183 (63.3%)
Total Cholesterol	54 [47–62]	110 [89–141]	25 [18–35]	124 [83–196]

* *p* < 0.01; ** *p* < 0.001; *** *p* < 0.0001

Shock/vasopressor requirement was significantly higher in low cholesterol group in both cohorts(*p* < 0.0001)

Only HARP-2 patients with sepsis are included

Values are presented as *n* (%) or median [interquartile range]

### Association of statin therapy with mortality in patients with low baseline cholesterol

**Fig. 1:**
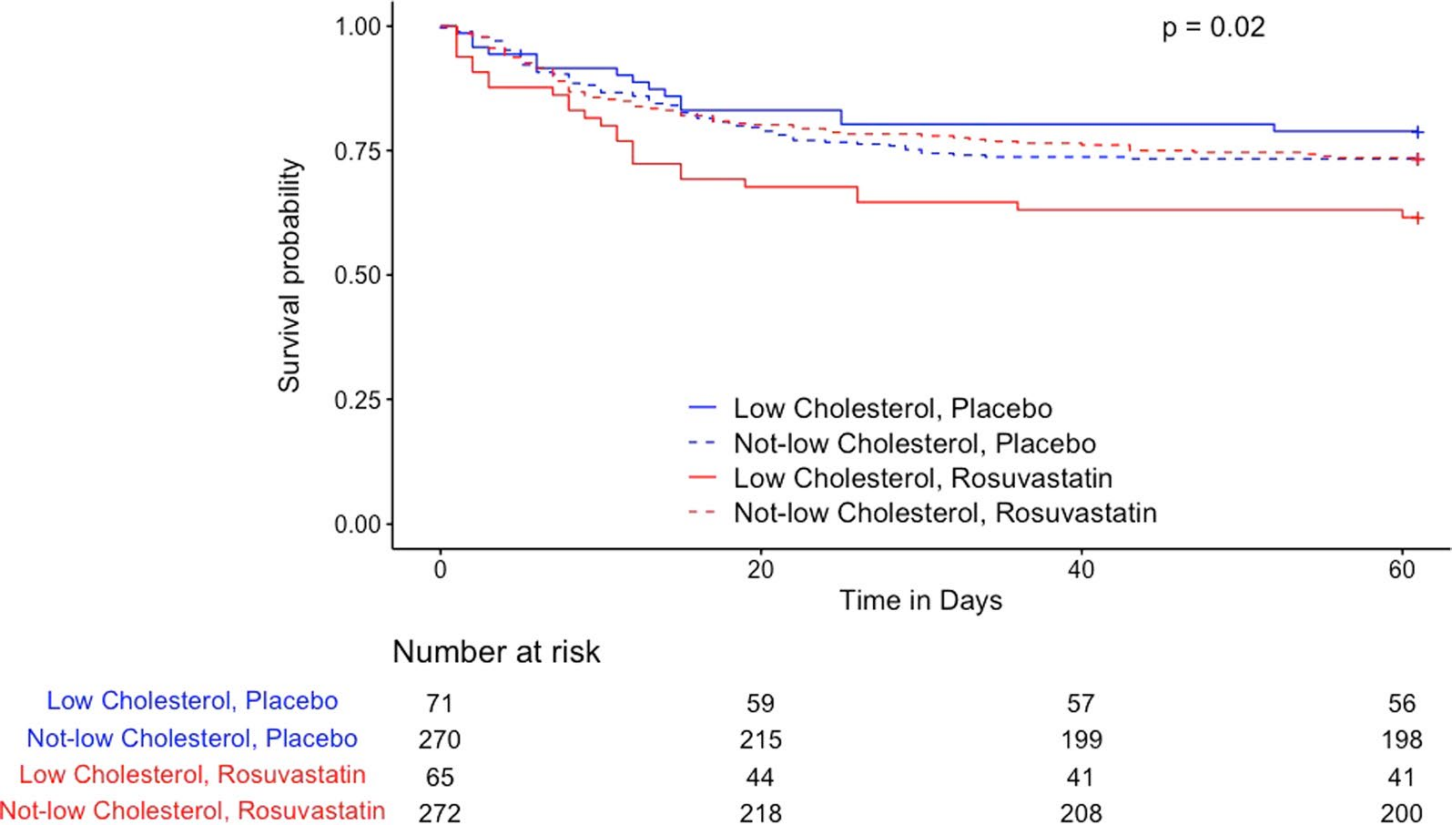
Kaplan–Meier Survival Analysis in SAILS (*N* = 678). “Low cholesterol” refers to the 1st cholesterol quartile. “Not-low cholesterol” refers to the 2nd–4th cholesterol quartiles. P value is for the interaction between cholesterol group and randomization to statin versus placebo according to the Cox Proportional Hazards model

The SAILS and HARP-2 trials were analyzed separately for association between low cholesterol and randomization to statin with survival. Mortality of these subjects is shown in Table 2, which stratifies individuals by cholesterol quartile and treatment groups. The 60-day mortality in the lowest cholesterol quartile of SAILS subjects randomized to rosuvastatin versus placebo was 37.8% versus 21.3% (OR 2.23, 95% CI 1.06–4.77, *p* = 0.02). Binary logistic regression for mortality at 60 days showed the interaction term for low cholesterol and randomization to statin was predictive of 60-day mortality (OR 2.57, 95% CI 1.14–5.85, *p* = 0.02). A post-hoc regression model including APACHE III score showed a strengthened interaction of cholesterol and rosuvastatin (*p* = 0.004) predicting mortality. Kaplan–Meier survival analysis (Fig. 1) showed reduced survival in low cholesterol subjects randomized to rosuvastatin when compared to the low cholesterol group which received placebo and the “not-low” cholesterol group which received rosuvastatin.

**Table 2:** 60-day mortality varies with baseline cholesterol level and treatment assignment in SAILS and HARP-2

Subgroup	Statin	Placebo	OR (Fisher’s)	Adjusted OR^1^	Interaction^1^	Fully Adjusted OR^2^	Fully Adjusted Interaction^2^
SAILS (rosuvastatin)							
Low Cholesterol	37.8%	21.3%	2.23 (95% CI 1.06–4.77, *p* = 0.02)	2.28 (95% CI 1.14–4.68, * p* = 0.02)	*p* = 0.02	3.35 (95% CI 1.55–7.59, * p* = 0.003)	*p* = 0.004
Not-low Cholesterol	26.2%	27.0%	0.96 (*p* = 0.92)	0.95 (*p* = 0.82)		0.93 (*p* = 0.75)	
HARP-2 (simvastatin)							
Low Cholesterol	31.9%	52.1%	0.44 (95% CI 0.17–1.07, *p* = 0.06)	0.48 (95% CI 0.20–1.14, *p* = 0.10)	*p* = 0.22	0.24 (95% CI 0.08–0.67, *p* = 0.009)	*p* = 0.045
Not-low Cholesterol	26.3%	26.9%	0.97 (*p* = 1.00)	0.93 (*p* = 0.80)		0.71 (*p* = 0.28)	

SAILS cohort *N* = 678 and HARP-2 *N* = 384 septic patients

Adjusted OR is for statin versus placebo group in binary logistic regression, after adjustment for age, gender and body mass index

^**1**^The cholesterol-statin interaction denotes the significance of the logistic regression interaction term between low cholesterol and randomization to statin therapy after adjustment for age, gender, and BMI

^2^This cholesterol-interaction indicates the significance of the logistic regression interaction term between low cholesterol and randomization to statin therapy after adjustment for age, gender, BMI, and APACHE II/III score. Inclusion of APACHE II/III score was not a prespecified analysis

**Fig. 2:**
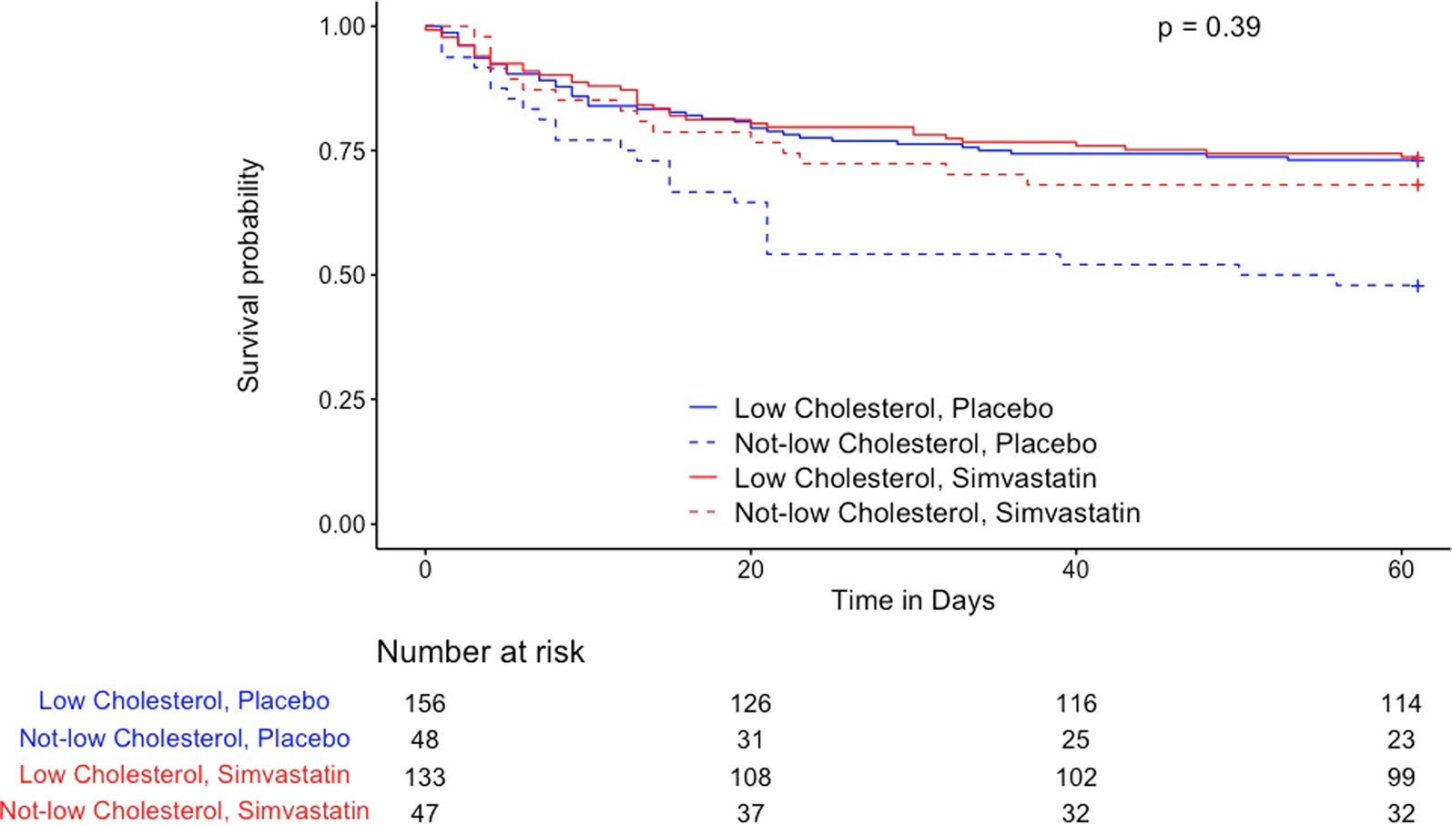
Kaplan–Meier Survival Analysis in HARP-2 (*N* = 384). “Low cholesterol” refers to the 1st cholesterol quartile. “Not-low cholesterol” refers to the 2nd–4th cholesterol quartiles. P value is for the interaction between cholesterol group and randomization to statin versus placebo according to the Cox Proportional Hazards model

In contrast to the effect of statin therapy in SAILS, HARP-2 patients with sepsis in the lowest cholesterol quartile who received simvastatin had a lower mortality than those in the placebo group although this did not reach statistical significance in this smaller cohort (31.9% vs. 52.1%, OR 0.44, 95% CI 0.17–1.07, *p* = 0.06). The interaction term between low cholesterol and simvastatin was not statistically significant (*p* = 0.22 by logistic regression, Table 2, and Kaplan–Meier results in Fig. 2). When all patients in HARP-2 are included rather than limiting to those with sepsis (*N* = 509), the findings were similar, with statistically significant lower mortality in patients with low cholesterol randomized to statin (28.6% vs. 53.1%, OR 0.36, 95% CI 0.16–0.78, *p* = 0.007). In this larger cohort, the interaction term between low cholesterol and simvastatin and 60-day mortality by logistic regression is significant (*p* = 0.047, Additional file 1: Table S1 and Additional file 2: Fig. S1). A post-hoc regression that additionally adjusted for APACHE II score again showed a significant interaction between low cholesterol and simvastatin treatment (*p* = 0.045, Table 2).

Given that the threshold for “low” cholesterol was different between the two cohorts (< 69 mg/dL in SAILS and < 44 mg/dL in HARP2), we performed a sensitivity analysis using additional cholesterol thresholds. As shown in Additional file 1: Table S2, the associations in the two cohorts are stable across various definitions of “low” cholesterol.

## Discussion

This study first investigated the association between total cholesterol levels with mortality and secondly the response to statin therapy in patients with ARDS and sepsis through a secondary analysis of the SAILS and HARP-2 trials. These cohorts had remarkably low baseline cholesterol levels, and low cholesterol correlated with increased illness severity in both cohorts. Subjects with lower cholesterol had higher mortality when randomized to receive rosuvastatin in the SAILS cohort. In contrast, fewer patients with sepsis and ARDS with low cholesterol randomized to simvastatin died in the HARP-2 trial compared to those who received placebo, though this did not reach statistical significance.

Median cholesterol level in our subsets with sepsis was 97 mg/dL in both SAILS and HARP-2, but perhaps more striking were the extreme values seen in the lowest quartile of total cholesterol, defined by < 69 mg/dL and < 44 mg/dL respectively. Higher rates of shock/vasopressor use are noted in HARP-2, which may indicate greater illness severity and thus contribute to the lower cholesterol levels seen in this trial. Reduced vascular tone has previously been associated with hypocholesteremia; however, the mechanism is not fully understood [21]. Of prior studies on statins in bacteremia or critical illness which reported cholesterol levels [22, 23], only Kruger et al*. *[24] presented comparably low median total cholesterol. Illness severity scores and rates of shock are higher in subjects in the lowest cholesterol quartile, suggesting that these values may be associated with greater severity of critical illness.

The direction of effect of statin therapy on mortality differed, with rosuvastatin associated with increased mortality in patients with low cholesterol, while simvastatin non-significantly decreased mortality in the same subset. This discrepancy between the trials might be due to differences in the properties and doses of these two statins. Rosuvastatin (administered at a daily dose of 20 mg in SAILS) was selected for SAILS because of its lower side effect profile and reduced incidence of drug-drug interactions. However, simvastatin (administered at a daily dose of 80 mg in HARP-2) had been previously proposed as a superior statin medication for ARDS and sepsis despite its lower potency as a lipid-lowering agent (rosuvastatin 10 mg is equivalent to simvastatin 72 mg and 77 mg for low-density and high-density lipoprotein reduction, respectively) [25–27] and was supported by data from early phase trials [4, 27, 28]. A large propensity score-matched study of mortality in sepsis reported that simvastatin and atorvastatin were protective, while rosuvastatin was not [29]. Simvastatin and atorvastatin have demonstrated greater antimicrobial activity than rosuvastatin against a broad range of common bacteria responsible for sepsis. These effects are seen at higher concentrations than were observed in plasma samples in SAILS, but some have proposed that accumulation in target tissues may explain clinical benefit of statins in bacterial infections in some cohorts [30]. LPS binding by lipoproteins is altered in sepsis, with a greater role for LDL sequestration of LPS under these conditions [31]. Because of the greater cholesterol-lowering potency of rosuvastatin, it is possible rosuvastatin is detrimental when cholesterol levels are already very low. Additionally, rosuvastatin is a hydrophilic molecule, while simvastatin is lipophilic; lipophilicity has previously been proposed as an important property for improving inflammatory biomarker profiles [8], and may affect drug concentration in target tissues. Data on statins point to pleiotropic effects in autoimmune/autoinflammatory diseases such as rheumatoid arthritis, multiple sclerosis, and acute graft-versus-host disease in bone marrow transplant recipients. Possible mechanisms include reduction in nuclear factor‐κB activation, increased release of interleukin-10, and reduced production of multiple pro-inflammatory cytokines as a downstream effect of hydroxymethyl-glutaryl coenzyme A reductase inhibition [32]. Robust prospective comparison of biological and clinical effects of different statin medications is limited outside of cardiovascular disease.

Though the original SAILS and HARP-2 trials did not show benefit for either statin in the overall population studied, there remains interest in studying statins further in ARDS subphenotypes, given the encouraging data for simvastatin in highly inflamed subjects in particular [7, 33]. Our analysis supports the possibility of benefit from simvastatin in subsets of severely ill patients with ARDS and suggests that low cholesterol could serve as an inclusion criteria for future trials with this agent if prospectively validated. Additionally, our secondary analysis which included all HARP-2 patients, regardless of sepsis, also showed reduced mortality. This finding suggests that simvastatin benefits in ARDS may not be limited to sepsis-related disease. In contrast, our findings suggest concomitant rosuvastatin should be used with caution in patients with low cholesterol and ARDS during their acute illness. Lipid levels should be measured in future trials of statin medications for critical illness so that safety and efficacy can be further evaluated in cholesterol subgroups.

Our study has methodological strengths, including the inclusion of two independent randomized, controlled trial cohorts, which allows assessment of statin impact in a low cholesterol group without concern for confounding. Because the statin medications utilized in SAILS and HARP-2 were different, we have been able to capture within-class differences in effects of statin medications in ARDS. While the association between low cholesterol and bad outcomes in sepsis has been reported, consideration of total cholesterol as a potential predictor of response to statins has not been studied before in ARDS to our knowledge, and helps inform an area with plausible biological relevance.

This study also has some weaknesses. First, despite including large RCT data, there was insufficient power to detect mortality differences which could be clinically significant. However, several models confirmed the mortality differences reported for low cholesterol subjects by treatment arm, and our observations of increased illness severity in low cholesterol subjects were also statistically significant by multiple tests. Secondly, the threshold for the lowest quartile differed in SAILS and HARP-2, and we did not define an optimal cholesterol threshold in this study. Third, cholesterol was not measured on fresh samples, but on stored aliquots years after study completion, and using two different assays for the two trials. We do note that our median values were similar not only to each other, but also to those described in the ANZ-STATInS trial [24]. Nonetheless, prospective validation of these findings and establishment of a threshold in future studies of statins in ARDS would be helpful, in part because cholesterol is readily measured in any clinical laboratory. Fourth, the results in this analysis do not imply a causal relationship between cholesterol and mortality in these populations, and rather describe an association which may represent an epiphenomenon. Lastly, correlation of total cholesterol at study enrollment with illness severity and mortality was not a prespecified endpoint for the SAILS or HARP-2 trials; this leads to the risk of type 1 error inherent in all secondary analyses.

## Conclusions

In conclusion, absolute values of cholesterol are low in these two ARDS cohorts, and the lowest levels of cholesterol are associated with greater severity of illness. There were differential effects of rosuvastatin and simvastatin in ARDS subjects with low cholesterol. We found rosuvastatin was associated with higher mortality in this subgroup, though simvastatin seems safe in these patients. Patients with low total cholesterol should be included in future trials of simvastatin as a treatment in ARDS.

## Supplementary Information

**Additional file 1. Table S1:** 60-day mortality for all subjects (with or without sepsis) in HARP-2. **Table 2:** Sensitivity analysis of morbidity and mortality by multiple cholesterol thresholds for subjects with sepsis-associated ARDS in SAILS and HARP-2.

**Additional file 2. Fig S1:** Survival curves for all subjects (with or without sepsis) in HARP-2 (N = 509). “Low cholesterol” refers to the 1st cholesterol quartile. “Not-low cholesterol” refers to the 2nd–4th cholesterol quartiles. P value is for the interaction between cholesterol group and randomization to statin versus placebo according to the Cox Proportional Hazards model.

## Data Availability

The datasets used and/or analyzed during the current study are available from the corresponding author on reasonable request. Data from this article have been presented in part at the American Thoracic Society Conference in 2020 (May 15–20, 2020) [34] and 2021 (May 14–19, 2021) [35].
