# Supplementary material for: Effect of total cholesterol and statin therapy on mortality in ARDS patients: a secondary analysis of the SAILS and HARP-2 trials
Source: Crit Care. 2023 Mar 28;27:126. doi: 10.1186/s13054-023-04387-9 (PMC10053133; doi:10.1186/s13054-023-04387-9)
Supplement: Supplementary file 1 — Additional file 1. Table S1: 60-day mortality for all subjects (with or without sepsis) in HARP-2. Table 2: Sensitivity analysis of morbidity and mortality by multiple cholesterol thresholds for subjects with sepsis-associated ARDS in SAILS and HARP-2. [file 13054_2023_4387_MOESM1_ESM.docx]

**Supplementary Appendix:**

**Supplementary Table 1: 60-day mortality for all subjects (with or without sepsis) in HARP-2**

| HARP-2 (simvastatin) | Statin | Placebo | OR (Fisher’s) | Adjusted OR | Interaction |
| --- | --- | --- | --- | --- | --- |
| Low Cholesterol | 28.6% | 53.1% | 0.36 (95% CI 0.16-0.78, p<0.01) | 0.38 (95% CI 0.17-0.80, p=0.01) | p=0.047 |
| Not-low Cholesterol | 24.2% | 25.0% | 0.96 (p=0.91) | 0.95 (p=0.83) |  |

N = 509

Adjusted OR is for statin versus placebo group in binary logistic regression, after adjustment for age, gender and body mass index.

The cholesterol-statin interaction denotes the significance of the logistic regression interaction term between low cholesterol and randomization to statin therapy after adjustment.

**Supplementary Table 2: Sensitivity analysis of morbidity and mortality by multiple cholesterol thresholds for subjects with sepsis-associated ARDS in SAILS and HARP-2**

|  | **Cholesterol <44 mg/dL (HARP-2 cutoff)** | **Pooled 1^st^ cholesterol quartile cutoff <62 mg/dL^1^** | **Cholesterol <69 mg/dL (SAILS cutoff)** | **Optimized cutoff of cholesterol <79 mg/dL^2^** |
| --- | --- | --- | --- | --- |
| **APACHE II*** | p=0.95 | p=0.97 | p=0.85 | p=0.35 |
| **APACHE III**** | p=0.004 | p<0.001 | p<0.001 | p<0.001 |
| **SOFA*** | p<0.001 | p=0.001 | p=0.001 | p<0.001 |
| **Shock/ Vasopressors** | OR 3.87, 95% CI 2.39-6.50, p<0.001 | OR 2.69, 95% CI 1.93-3.80, p<0.001 | OR 2.36 95% CI 1.74-3.22, p<0.001 | OR 2.20, 95% CI 1.66-2.92, p<0.001 |
| **60-day mortality** | OR 1.70, 95% CI 1.13-2.54, p=0.008 | OR 1.13, 95% CI 0.84-1.54, p=0.41 | OR 1.34, 95% CI 0.99-1.80, p=0.051 | OR 1.76, 95% CI 1.32-2.33, p=<0.001 |

N = 1062, unless indicated by asterisk.

Wilcoxon rank-sum test was used for to assess relationship of APACHE II, APACHE III, and SOFA with the presented cholesterol thresholds. Fisher’s exact test was used for shock/vasopressors and 60-day mortality.

^1^The pooled 1^st^ cholesterol quartile cutoff of <62 mg/dL was determined by aggregating all patients in SAILS and HARP-2.

^2^The optimized cutpoint of <79 mg/dL was determined by maximizing sensitivity and specificity for 60-day mortality.

*Data only available for HARP-2, N = 678

**Data only available for SAILS, N = 384

**Supplementary Figure 1: Survival curves for all subjects (with or without sepsis) in HARP-2**

N = 509

“Low cholesterol” refers to the 1^st^ cholesterol quartile. “Not-low cholesterol” refers to the 2^nd^-4^th^ cholesterol quartiles.

P value is for the interaction between cholesterol group and randomization to statin versus placebo according to the Cox Proportional Hazards model.
