# Supplementary figures and images for: Effect of total cholesterol and statin therapy on mortality in ARDS patients: a secondary analysis of the SAILS and HARP-2 trials
Source: Crit Care. 2023 Mar 28;27:126. doi: 10.1186/s13054-023-04387-9 (PMC10053133; doi:10.1186/s13054-023-04387-9)

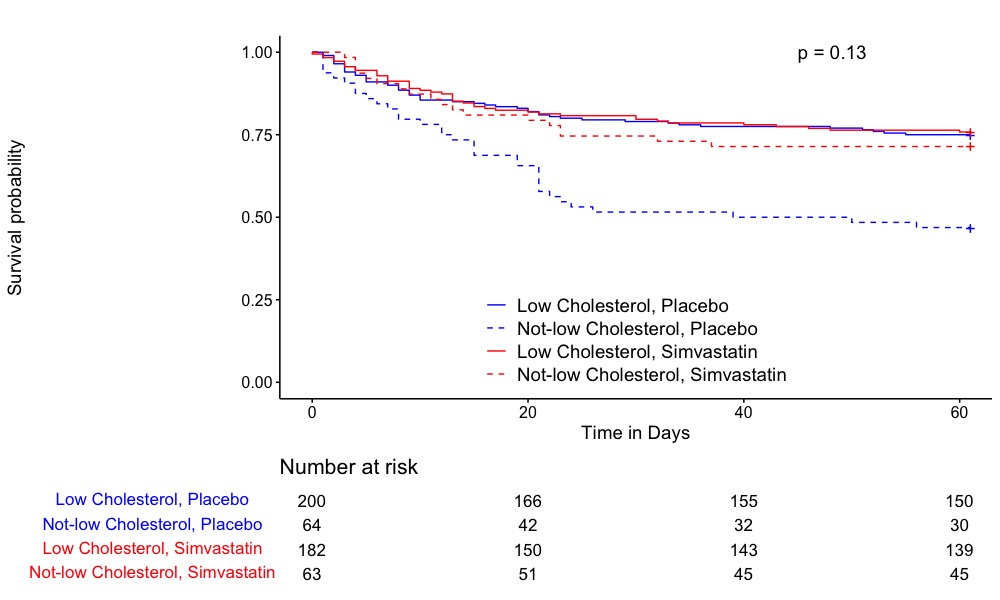

Supplement: Supplementary file 2 — Additional file 2. Fig S1: Survival curves for all subjects (with or without sepsis) in HARP-2 (N = 509). “Low cholesterol” refers to the 1st cholesterol quartile. “Not-low cholesterol” refers to the 2nd–4th cholesterol quartiles. P value is for the interaction between cholesterol group and randomization to statin versus placebo according to the Cox Proportional Hazards model. [file 13054_2023_4387_MOESM2_ESM.jpeg]
